# Cnf1 Variants Endowed with the Ability to Cross the Blood–Brain Barrier: A New Potential Therapeutic Strategy for Glioblastoma

**DOI:** 10.3390/toxins12050291

**Published:** 2020-05-04

**Authors:** Andrea Colarusso, Zaira Maroccia, Ermenegilda Parrilli, Elena Angela Pia Germinario, Andrea Fortuna, Stefano Loizzo, Laura Ricceri, Maria Luisa Tutino, Carla Fiorentini, Alessia Fabbri

**Affiliations:** 1 Department of Chemical Sciences, University of Naples Federico II, Complesso Universitario M. S. Angelo, Via Cintia, 80126 Napoli, Italy; andrea.colarusso@unina.it (A.C.); erparril@unina.it (E.P.); tutino@unina.it (M.L.T.); 2 Istituto Superiore di Sanità, Viale Regina Elena 299, 00161 Rome, Italy; zaira.maroccia@iss.it (Z.M.); elena.germinario@iss.it (E.A.P.G.); andrea.fortuna@iss.it (A.F.); stefano.loizzo@iss.it (S.L.); laura.ricceri@iss.it (L.R.); carla.fiorentini@iss.it (C.F.); 3 Association for Research on Integrative Oncological Therapies (ARTOI), 00165 Rome, Italy

**Keywords:** glioblastoma, drug discovery, cytotoxic necrotizing factor type 1, protein purification, recombinant protein production

## Abstract

Among gliomas, primary tumors originating from glial cells, glioblastoma (GBM) identified as WHO grade IV glioma, is the most common and aggressive malignant brain tumor. We have previously shown that the *Escherichia coli* protein toxin cytotoxic necrotizing factor 1 (CNF1) is remarkably effective as an anti-neoplastic agent in a mouse model of glioma, reducing the tumor volume, increasing survival, and maintaining the functional properties of peritumoral neurons. However, being unable to cross the blood–brain barrier (BBB), CNF1 requires injection directly into the brain, which is a very invasive administration route. Thus, to overcome this pitfall, we designed a CNF1 variant characterized by the presence of an N-terminal BBB-crossing tag. The variant was produced and we verified whether its activity was comparable to that of wild-type CNF1 in GBM cells. We investigated the signaling pathways engaged in the cell response to CNF1 variants to provide preliminary data to the subsequent studies in experimental animals. CNF1 may represent a novel avenue for GBM therapy, particularly because, besides blocking tumor growth, it also preserves the healthy surrounding tissue, maintaining its architecture and functionality. This renders CNF1 the most interesting candidate for the treatment of brain tumors, among other potentially effective bacterial toxins.

## 1. Introduction

Gliomas are primary tumors occurring in the central nervous system (CNS) that arise from glial cells. Glioblastoma (GBM; World Health Organization grade IV glioma) [1] represents the most malignant form, being highly invasive and not responsive to standard treatments that include surgical resection of the tumoral mass followed by combined radio- and chemo-therapy. Therefore, the life expectancy after diagnosis is very poor [2]. 

We have previously shown that the *Escherichia coli* protein toxin cytotoxic necrotizing factor 1 (CNF1) is remarkably effective as an anti-neoplastic agent in a mouse model of glioma, reducing the tumoral mass, increasing the survival, and maintaining the functional properties of peritumoral neurons [3,4]. CNF1 specifically modulates Rho GTPases, thus affecting fundamental cellular processes that may induce different outcomes in different cell types. In fact, besides triggering an epithelial-mesenchymal transition in transformed epithelial cells where the movement and proliferating activity of the toxin are increased [3], CNF1 inhibits the growth of glioma cells that finally undergo death. Its detrimental activity on GBM cells is probably due to its ability to block cytodieresis in proliferating cells, leading them to senescence and death, whereas the beneficial activity on peritumoral neurons is still under investigation but seems to rely on the enhancement of synaptic plasticity and the preservation of functional attributes. These abilities point at CNF1 as a possible new strategy for the treatment of brain tumors. 

However, being unable to cross the blood–brain barrier (BBB), CNF1 requires an intracerebral injection, as evidenced in all studies so far conducted using CNF1 on mouse models for CNS pathologies, including Rett syndrome (RTT) [5,6,7], Alzheimer’s disease [8], epilepsy [9] as well as GBM [4,10,11]. Therefore, considering the invasive route of administration, the difficulty to propose the toxin as an innovative therapy prompted us to seek more tolerable delivery systems. To address this point, we have designed and produced a CNF1 variant that might be endowed with the ability to cross the BBB when intravenously (*iv*) injected (An2-CNF1-H8). Its main feature consists of the addition of an N-terminal Angiopep-2 (An2) peptide which should promote receptor-mediated transcytosis [12]. The recombinant protein was completed by the addition of a C-terminal His-tag (H8) to be used for the recombinant toxin purification. The demonstration that An2-CNF1-H8 preserves the original activity profile of the wild-type (*wt*) protein in different cell types, would also reinforce the hypothesis of CNF1 as a new candidate drug for the treatment of other disorders, such as RTT and mitochondrial encephalomyopathies. In particular, GBM cells have been the recipient for a more accurate investigation, propaedeutic to in vivo studies. 

## 2. Results

### 2.1. Design and Production of a CNF1 Variant Bearing the Potentiality to Cross the BBB When Peripherally Injected

**Figure 1:**
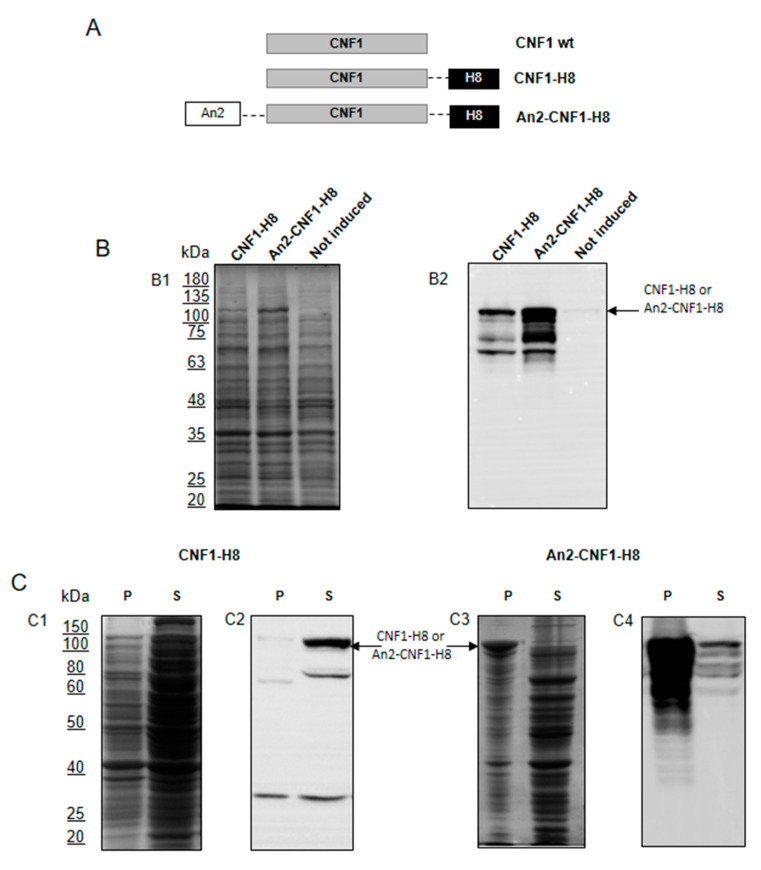
Design, expression, and solubility of cytotoxic necrotizing factor 1 (CNF1) variants. (**A**) Scheme of engineered CNF1 constructs presenting the following N-terminal and C-terminal tags: Angiopep-2 (An2), spaced by the GGSSRSS linker; 8xHis fused to a Tobacco Etch Virus recognition site (H8). (**B**) Recombinant protein expression levels in *E. coli* BL21 (DE3). Total cellular extracts obtained at 15 °C after 18 h induction with 0.5 mM IPTG were separated by 10% SDS-PAGE and analyzed either by Coomassie staining (**B1**) or western blot (**B2**) using a monoclonal anti-CNF1 antibody (NG8). Induced cells expressing CNF1-H8; induced cells expressing An2-CNF1-H8; not induced recombinant cells harboring pET40b-*cnf1-h8*. (**C**) Evaluation of CNF1 solubility in *E. coli* extracts. After cellular lysis, insoluble (P) and soluble (S) fractions were analyzed. The first two panels are relative to the solubility assay concerning CNF1-H8 expression carried out using SDS-PAGE (**C1** and **C3**) and western blot (**C2** and **C4**). The third and fourth images represent the SDS-PAGE and western blot relative to An2-CNF1-H8 solubility, respectively.

We have previously demonstrated that a carboxy terminally his-tagged version of CNF1 (CNF1-H8) can be efficiently expressed in and purified from recombinant *E. coli* BL21(DE3) cells [13]. In this study, we attempted the production of a slightly modified version of the same toxin harboring an N-terminal peptide, named Angiopep-2 (An2), and capable of interacting with low-density lipoprotein receptor-related protein-1 (LRP1). This receptor is known to be involved in the transcytosis of the brain delivery vector An2 [12]. This new version of the protein was named An2-CNF1-H8, as it still possessed a C-terminal polyhistidine tag (Figure 1A). The recombinant expressions of both CNF1-H8 and An2-CNF1-H8 were carried out at a low temperature of 15 °C, as previously reported [13]. The analysis of total cellular extracts obtained at the end of the bacterial growths indicated that an overexpression was clearly visible for both the constructs via SDS- polyacrylamide gel electrophoresis (PAGE) (Figure 1(B1)) and western blot (Figure 1(B2)). It is worth noting that the addition of the N-terminal An2 peptide led to a significantly higher production of the protein in comparison with the CNF1-H8 (Figure 1B). This outcome might be due to either a more efficient translation initiation or an increased half-life of the protein. Nevertheless, in terms of recombinant production of An2-CNF1-H8, this boost proved to be unfruitful, as the protein mainly accumulated as insoluble aggregates. This result is depicted in Figure 1C, where soluble (S) and insoluble (P) fractions, collected after cellular disruption, were analyzed via SDS-PAGE (Figure 1(C1,C3)) and western blot (Figure 1(C2,C4)). In the case of CNF1-H8 bearing bacteria, the recombinant product mainly accumulated as a soluble protein, as observable in the left part of Figure 1C. Conversely, *E. coli* synthesizing An2-CNF1-H8 suffered from the formation of inclusion bodies, distinguishable both in the Coomassie-stained gel and in western blot in the right part of Figure 1C. Despite a considerable loss of An2-CNF1-H8 as insoluble aggregates, its recovery in the cellular soluble fraction was still feasible using a previously established procedure [13].

### 2.2. The An2-CNF1-H8 Variant Preserves the Original Activity Profile of wt CNF1 in HEp-2 Cells

**Figure 2:**
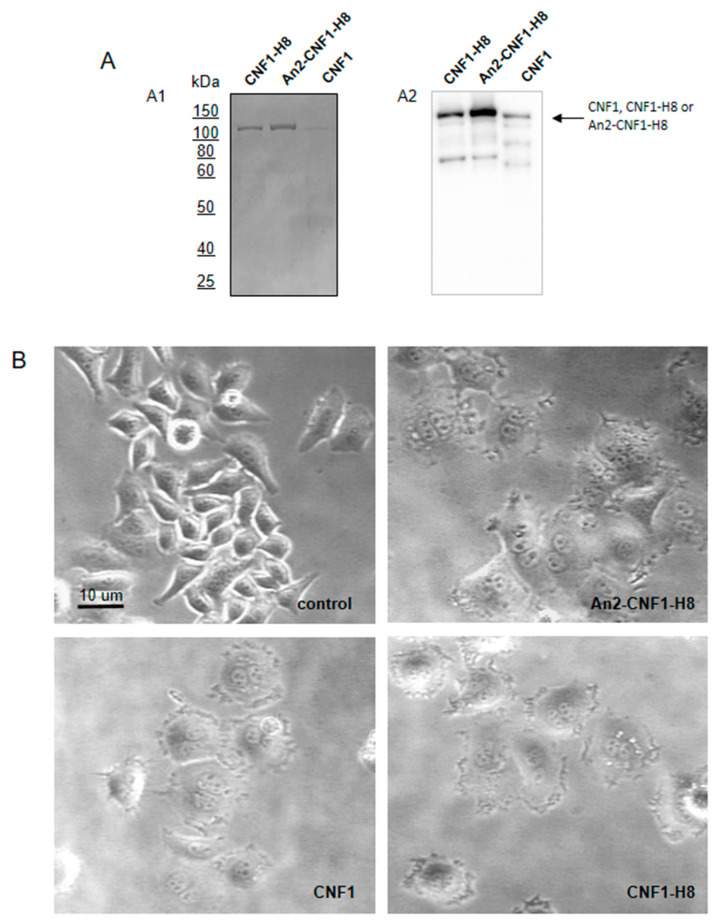
CNF1 variants activity. (**A**) SDS-polyacrylamide gel (**A1**) and western blot (**A2**) of CNF1 variants. (**B**) Phase-contrast micrographs of HEp-2 cells treated with CNF1 variants, showing the multinucleating/ruffling effects. Scale bar: 10 μm.

The activity of the An2-CNF1-H8 variant was first tested on HEp-2 cells. The first set of experiments was carried out to verify whether the An2-CNF1-H8 variant could act in the same vein of CNF1. Therefore, to address this point, we used HEp-2 cells, the best characterized and responsive cell model for CNF1. CNF1-H8, and *wt* CNF1 were employed as controls in all the experiments. Once assessed that the An2-CNF1-H8 sample was more concentrated than *wt* CNF1 and CNF1-H8, as evidenced by polyacrylamide gel and western blot analysis (Figure 2A), we performed a titration experiment to find the lowest concentration inducing CNF1-like effects, i.e., multinucleation and ruffling (Figure S1). We found that the morphological effects induced by the An2-CNF1-H8 variant were comparable to that of *wt* CNF1 and obtained in the same concentration range (Figure 2B). 

### 2.3. The An2-CNF1-H8 Variant Enters the Cells in the Same Vein of wt CNF1

**Figure 3:**
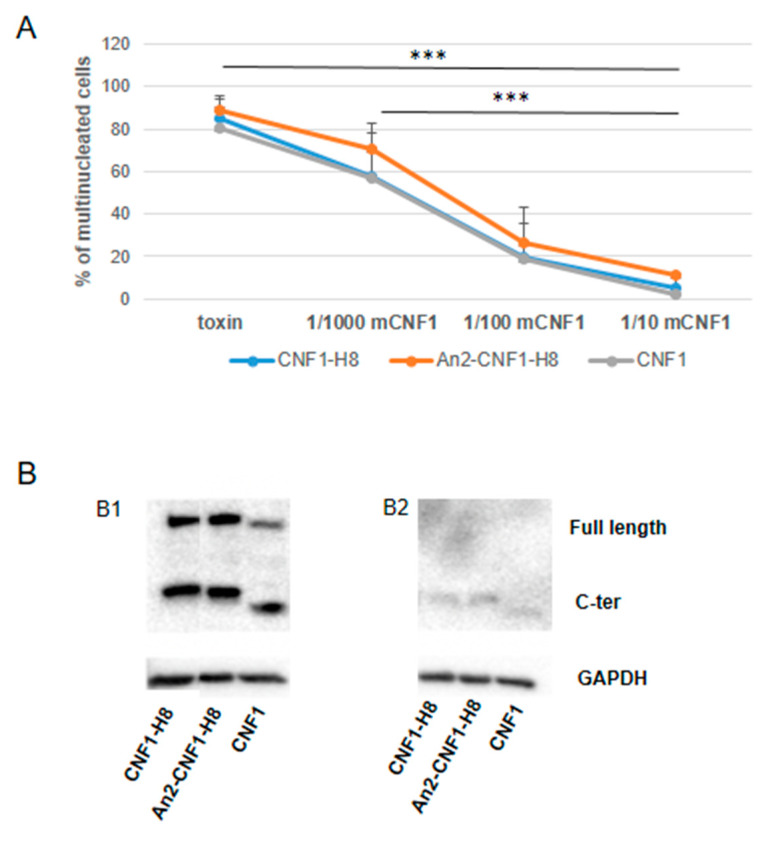
Entry of CNF1 variants into HEp-2 cells. (**A**) Graph showing the inhibition of variants entry by mCNF1. (**B**) Western blot analysis of HEp-2 cells treated with An2-CNF1-H8 and its controls (CNF1-H8 and *wt* CNF1) and stained with an antibody against CNF1, normalized as a function of GAPDH. Note that all the toxins are found as full length and 55 kDa C-ter portions after 24 h treatment (**B1**). Over a longer period of time, that is 48 h of treatment, washing to eliminate any remaining molecules in the culture medium, and further culture for 24 h, the full-length portions completely disappeared while the 55 kDa C-ter portions were present at a much lower concentration (**B2**). All in all, these results point at the fact that An2-CNF1-H8 follows the same route of entry of *wt* CNF1. Variants and *wt* CNF1 were used at 10^−10^ M. Data on the graph represent the mean ± SEM from at least three independent experiments. *** *p* < 0.001.

To investigate whether the An2-CNF1-H8 variant binds to the same receptor engaged by the *wt* CNF1 or if it can enter via alternative pathways or receptors, we performed experiments in which we challenged the non-catalytic mutant CNF1-C866S (mCNF1, which maintains the ability to bind to the receptor but does not possess the enzymatic activity) with the CNF1 variant. As shown in Figure 3A, as the dose of mCNF1 increases, the An2-CNF1-H8 variant is inhibited, i.e., the percentage of multinucleated cells is decreased to zero, proving the engagement of the same receptor used by *wt* CNF1, at least in HEp-2 cells.

Then, to verify if the variant enters the cells via the canonical pathway of CNF1 endocytosis, being processed to release the C-terminal catalytic fragment (CNF1-C-ter) into the cytoplasm, HEp-2 cells were treated with An2-CNF1-H8 at the concentration of 10^−9^ M and lysed after 24 h. As evidenced in Figure 3(B1), the CNF1-C-ter portion of the variant was present in the cytoplasm, proving the entry through the classical CNF1 pathway. At the same time, we checked if all the administrated toxin was internalized over a longer period of time. To address this last question, we washed the cells with fresh medium after 48 h of treatment, to eliminate any remaining molecules left in the culture medium. The cells were then cultured for further 24 h before being lysed and subjected to western blot using the anti-CNF1 against the CNF1-C-ter. It should be noted that in these conditions (Figure 3(B2)) the whole CNF1 completely disappeared and the C-ter parts were present at a much lower concentration if compared to cells exposed for 24 h only. This indicates that, not only all the proteins are processed by freeing the toxin C-ter portion in the cytoplasm, but also that an amount of C-ter portion undergoes an elimination process.

### 2.4. Activities of the An2-CNF1-H8 Variant on the Human Brain Endothelial Cell Line HBEC-5i

**Figure 4:**
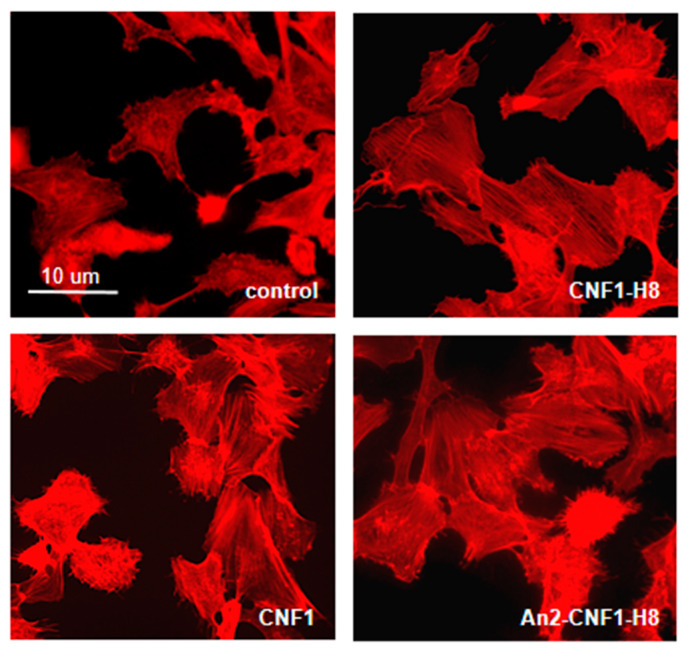
An2-CNF1-H8 activity on endothelial cells. Fluorescence micrographs of HBEC-5i cells treated with the An2-CNF1-H8 variant and its controls. Note the same actin architecture in cells exposed to the *wt* CNF1 and to the variants, indicating the same activity. Scale bar: 10 μm.

The variants were built to find an inoculation system less invasive than the intracerebroventricular injection, such as the *iv* administration. Since *iv*-injected toxins need to act on endothelial cells in order to cross the BBB, we verified the activity of the CNF1 variant on the human brain endothelial cell line HBEC-5i. Interestingly, HBEC-5i cells highly express the LRP1 receptor for An2 and, therefore, we analyzed whether the variant could enter in this cellular system more easily than in the others. As reported in Figure 4, the An2-CNF1-H8 variant induces modification of the actin architecture on HBEC-5i cells comparable to that of *wt* CNF1, also in terms of fluorescence intensity (Figure S2). Moreover, competition experiments with the mutant CNF1 C866S were carried out in this endothelial cell line as in HEp-2 cells, and the results were overlapping (not shown). Therefore, our results demonstrate that An2-CNF1-H8 behaves as the *wt* CNF1 even in HBEC-5i cells. On the other hand, our preliminary in vivo experiments show that when mice were *iv* inoculated with the An2-CNF1-H8 variant, spinophilin amount increased in the hippocampus after 4 h (data not shown) as well as 7 days from inoculation (Figure S3), suggesting that the variant could pass the BBB. 

### 2.5. Activities of the An2-CNF1-H8 Variant on the Human GBM Cells 

**Figure 5:**
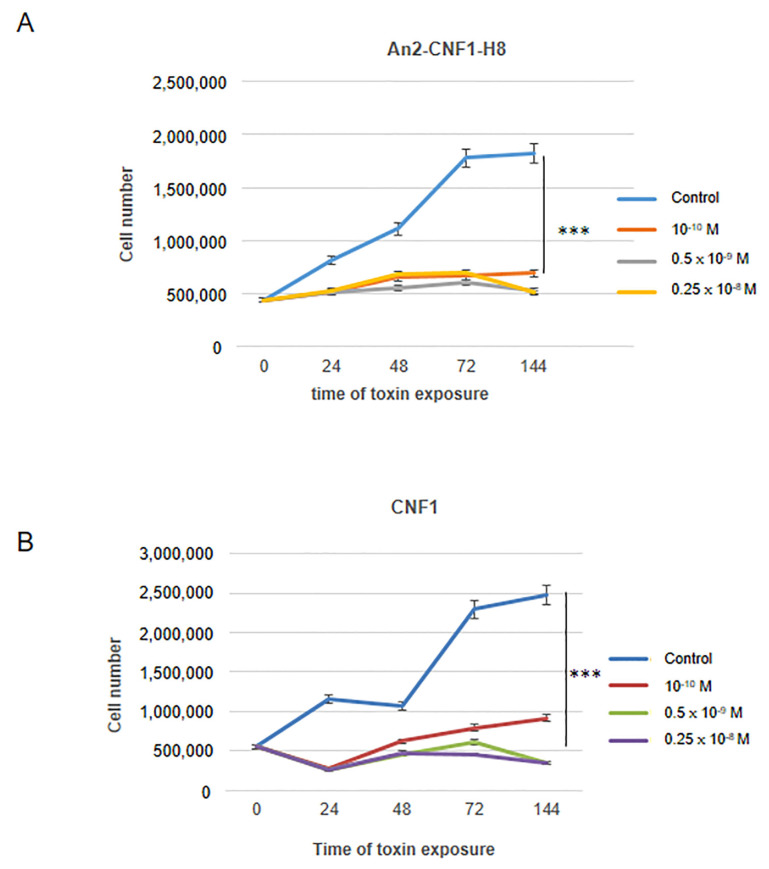
An2-CNF1-H8 impairs growth in U87MG GBM cells. (**A** and **B**) Graphs showing cell growth impairment by An2-CNF1-H8 (**A**) and CNF1 (**B**) on U87MG GBM cells. Note that, whereas controls cells increase in cell number in the function of time, cells treated with An2-CNF1-H8 and CNF1 stopped growing. Data on the graph represent the mean ± SEM from at least three independent experiments. *** *p* < 0.001.

**Figure 6:**
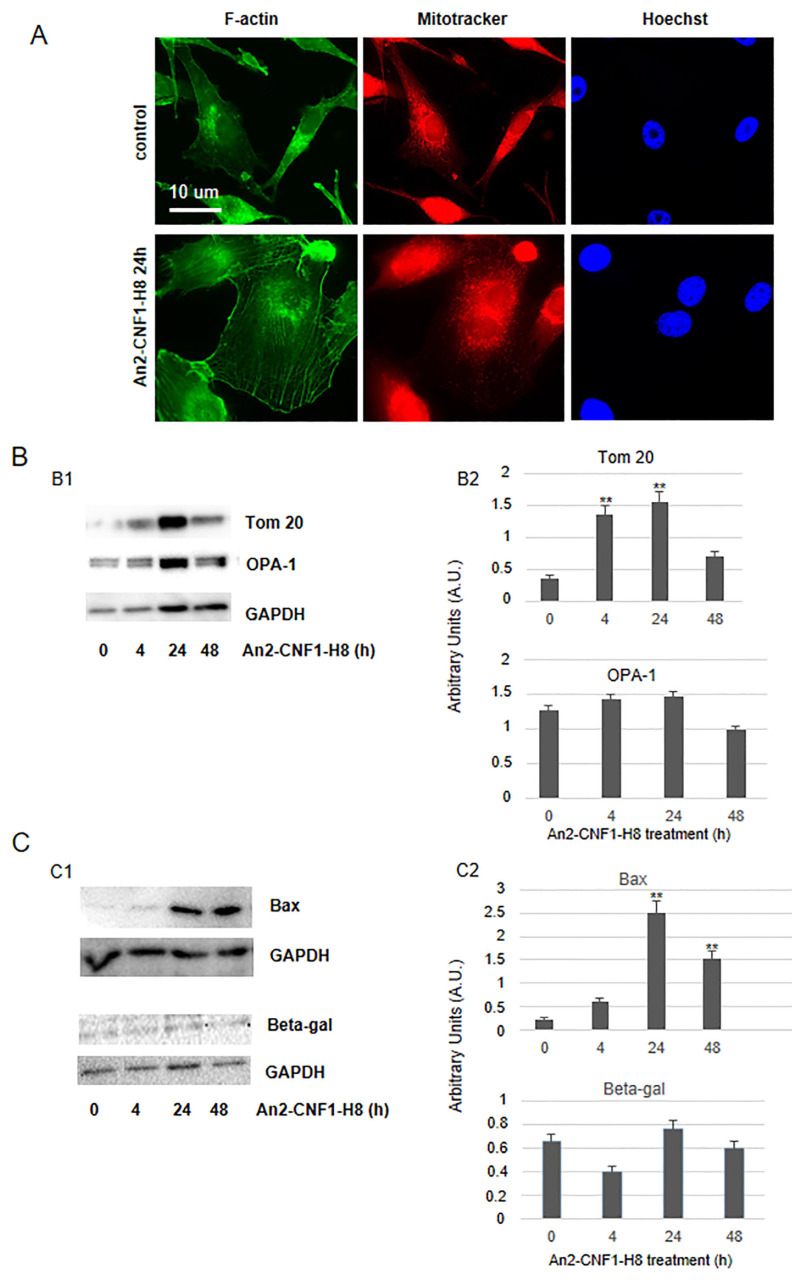
Mitochondrial impact and toxicity of An2-CNF1-H8 on U87MG cells. (**A**) Fluorescence micrographs of U87MG cells treated with An2-CNF1-H8 and stained with phalloidin, to detect F-actin, Mitotracker, to detect mitochondria and Hoechst 33342 to stain nuclei. Note that the variant impacts on mitochondria that appear slightly fragmented and scattered throughout the cell body with respect to controls. (**B** and **C**) Western blot of U87MG cells treated with An2-CNF1-H8 and graph analyses, showing that (**B1** and **B2**) Tom20 was transiently increased, whereas OPA-1 protein was not modified by the An2-CNF1-H8; (**C1** and **C2**) the pro-apoptotic Bax protein, involved in mitochondrial outer membrane permeabilization, was increased whereas the senescence marker beta-gal remained unvaried. Proteins were normalized as a function of GAPDH. ** *p* < 0.01. Scale bar: 10 μm.

Since CNF1 has been reported to be remarkably effective as an antineoplastic agent in a mouse model of GBM, and to induce toxicity in GBM cells obtained from surgical specimens (WHO grade IV) [4,10,11], we verified whether the An2-CNF1-H8 variant could be effective on a human GBM cell line, the U87MG cells. We performed a cell growth analysis and, as shown in Figure 5A, whereas control cells underwent an increase in cell number over time, although characterized by a biphasic growth possibly due to depletion of some rate-limiting resource, cells treated with different concentrations of An2-CNF1-H8 stopped growing, starting from 24 h of exposure. The same result was obtained with the *wt* CNF1 (Figure 5B). A morphological analysis showed that the An2-CNF1-H8 variant could induce F-actin modification, in term of stress fibers and ruffles, and multinucleation. Interestingly, it caused an increase in the number of mitochondria but a reduction in their size, with respect to controls (Figure 6A). Western blot analysis showed that Tom20, a component of the translocase of the outer mitochondrial membrane (Tom), the main mitochondrial entry gate for nuclear-encoded proteins, was transiently increased following treatment with An2-CNF1-H8, with the maximum increment at 24 h. After 48 h of An2-CNF1-H8 treatment, Tom20 expression returned similar to the control level (Figure 6B). The amount of OPA-1 protein, involved in mitochondrial fusion, was not modified by the variant An2-CNF1-H8 (Figure 6B), as reported for *wt* CNF1 in other model systems [9,14]. We wondered whether the An2-CNF1-H8 variant stimulates senescence or apoptosis to cause cell number reduction. We then analyzed Bax, a pro-apoptotic protein, and found that An2-CNF1-H8 variant treatment induced an increase in Bax concentration starting from 24 h of exposure (Figure 6C), thus suggesting an involvement of apoptosis in the variant killing activity. However, we failed to observe the activation of caspase-3 (data not shown). On the other hand, western blot analysis of the senescence-associated beta-galactosidase (beta-gal) showed no significant differences (Figure 6C).

## 3. Discussion

We previously reported an efficient strategy for the production and purification of the CNF1 toxin as a his-tagged recombinant protein (CNF1-H8) from *E. coli* extracts [13]. In the present manuscript, we describe the production and purification of the An2-CNF1-H8 variant, a CNF1 variant displaying an N-terminal Angiopep tag potentially endowed with the ability to allow BBB crossing. 

The recombinant production of the An2-CNF1-H8 variant was approached following the protocol optimized for CNF1-H8. It turned out that the amount of recombinant An2-CNF1-H8 variant was significantly higher, but it largely accumulated as inclusion bodies, although the production was carried out at 15 °C by using only 0.5 mM inducer. Lowering of the production temperature can be a successful approach to get the soluble product when the protein has the tendency to accumulate as inclusion bodies, as in the case of CNF1-H8 [13]. However, the higher production yields achieved for An2-CNF1-H8 shifted the equilibrium towards the formation of insoluble aggregates. Nevertheless, its purification from the soluble extracts in native conditions was still feasible. Once purified, the cellular activity of the An2-CNF1-H8 variant was compared to that of *wt* CNF1 and it turned out that cellular effects completely overlapped, causing multinucleation and actin cytoskeleton changes in the same concentration ranges. 

It is interesting to note that the route of entry into cells followed by the An2-CNF1-H8 variant is the same used by the *wt* CNF1. It is known that CNF1 binds to its receptor [15,16], enters endocytic vesicles by receptor-mediated endocytosis and is routed to the endosomal compartment [17] where its catalytic domain (55-kDa fragment) is transferred into the cytosol [18]. The mutant toxin CNF1 C866S, herein indicated as mCNF1, devoid of enzymatic activity [19] was able to compete for the binding to the receptor with the An2-CNF1-H8 variant inhibiting its multinucleating activity. Besides, a 55-kDa fragment was recorded into the cytosol. All these findings indicate that the variant behaves in the same way of *wt* CNF1, demonstrating that the addition of An2 and H8 at the N- and C-terminal end, respectively, did not compromise its activity on epithelial cells, although further studies on neuronal cell models should also be performed. When endothelial cells were exposed to An2-CNF1-H8, a typical CNF1-induced actin rearrangement was observed, indicating that the variant entered into this cell line and exerted its action. Since HBEC-5i cells highly express the LRP1 receptor for An2, we were expecting a better efficiency of An2-CNF1-H8 in its entry into this cell type with a consequent major effect on the targets. The observed actin rearrangement, however, was in line with what so far documented in other cell systems, underlying the necessity of further experiments to verify our hypothesis. It is interesting to note that *iv* inoculation of the An2-CNF1-H8 variant in mice can increase the amount of spinophilin in the hippocampus. This result, besides suggesting the ability of the variant to link its LRP1 receptor, represents an important step forward the demonstration of the An2-CNF1-H8 ability to cross the BBB. Further studies are needed, however, to confirm that An2-CNF1-H8 can actually pass through the barrier. 

Our previous studies disclosed the potentiality of CNF1 as a novel therapeutic strategy for the treatment of CNS tumors. Vannini and coworkers reported that CNF1 possesses a cytotoxic effect on a GBM cell line as well as on primary human GBM cells, also permitting a long-term survival in a murine GBM model [10]. Besides, CNF1 reduces GBM growth and saves the function and structure of neurons [4]. Our results demonstrate that the An2-CNF1-H8 variant exerts a cell growth arrest of U87MG GBM cells in the same vein of CNF1. From a morphological point of view, it is interesting to note that An2-CNF1-H8, although inducing typical CNF1 effects consisting of F-actin polymerization and multinucleation [20], impacts on mitochondria in a different way in comparison to data reported so far. On epithelial cells and fibroblasts, CNF1 induces mitochondria elongation [21,22] due, at least in epithelial cells, to inhibition of mitochondrial fission [14]. Here, we report that, in U87MG GBM cells, both the An2-CNF1-H8 variant and CNF1 (data not shown), induced fragmentation of mitochondria and did not vary the amount of OPA1 protein, involved in mitochondria fusion. However, the number of mitochondria seems to be increased in cells challenged with the variant with respect to control cells, as also suggested by the transient increase in Tom20, a component of the outer mitochondrial membrane [23]. The dynamic of mitochondria allows the cell to respond to its ever-changing physiological conditions and in this context, fragmented mitochondria can be found in damaged or quiescent cells, associated with functional defects or early in the apoptotic pathway [24]. For this reason, we analyzed some proteins of the apoptotic as well as of the senescence pathway. Our results demonstrate that the An2-CNF1-H8 variant increased the amount of the pro-apoptotic protein Bax, although we failed to find a concomitant activation of caspase-3. On the other hand, the level of the senescence-associated marker beta-gal was almost unchanged with respect to control cells. Further studies are needed to dissect the cellular pathway engaged by the An2-CNF1-H8 variant to arrest cell growth of GBM cells. In fact, senescence may be a late phenomenon, as reported by Vannini and coworkers [10] whereas caspases may be activated later in our model. Besides, we cannot rule out the hypothesis that necroptosis can occur in U87MG GBM cells exposed to the An2-CNF1-H8 variant [24]. It is worth noting that CNF1 is being increasingly recognized as a pro-carcinogenic agent in the intestine [3,25], indicating that the toxin behaves in two different manners in the intestine and in the brain. This is not surprising since bacterial toxins, by acting as signaling modifying agents, are profoundly influenced by several factors such as, for example, different environmental and cellular/tissue conditions. In this context, it is well established that CNF1 exerts beneficial effects in mice models of Rett Syndrome and Alzheimer once injected in the brain [6,8]. On the other hand, we have very recently reported that the CNF1 ability to promote carcinogenic traits in epithelial cells is dramatically influenced by the surrounding microenvironment as well as by the cell type [3]. In all the cell types studied so far, CNF1 causes mitochondrial elongation whereas we herein report that the CNF1 variant causes mitochondria fragmentation in GBM cells, reinforcing the emerging hypothesis that the target cell type plays a pivotal role in the response to an infectious agent [26]. This last finding on mitochondria highlights a new perspective for the understanding of CNF1 activity on GBM cells. 

Altogether, our results point at the An2-CNF1-H8 variant as a promising tool against GBM, preserving the same cytotoxic activities of CNF1 but being characterized by the potential ability to cross the BBB. 

## 4. Materials and Methods 

### 4.1. Production and Purification of CNF1 and CNF1 Variants

*cnf1-h8* and *an2-cnf1-h8* genes were expressed using pET40b plasmid. The preparation of pET40b-*cnf1-h8* has been previously described [13]. For the development of pET40-*an2-cnf1-h8*, the 5′ extremity of *cnf1-h8* was replaced with a newly synthesized one using NdeI/BglII double digestion. The new DNA fragment of 188 bp was purchased from Thermo Fisher Scientific (Thermo Fisher Scientific, Waltham, MA, USA) and encodes the An2 peptide (MGTFFYGGSRGKRNNFKTEEY) coupled to a linker (GGSSRSSS) that has already been used for the production of a GFP variant fused to An2 [27]. 

Both An2-CNF1-H8 and CNF1-H8 were produced in *E. coli* BL21(DE3) and purified using two chromatographic steps as reported [13]. For preliminary solubility analysis, the recombinant cellular pellets were re-suspended in 5 mL/g wet cell weight of Lysis buffer (50 mM TrisHCl pH 8.0, 150 mM NaCl, 20 mM Imidazole, 15% v/v glycerol) supplemented with the complete EDTA-free protease inhibitor cocktail (Roche, Basel, Switzerland). Afterward, the suspensions were sonicated (30 s cycles, 25% amplitude with 30 sec pauses between each cycle for a total process of 20 min at 4 °C) and the soluble and insoluble fractions were separated by centrifugation (13,000 g, 4 °C, 45 min). Then, the insoluble fractions were re-suspended in an equal volume of Lysis buffer as its relative soluble fraction and the total protein profiles were studied using 10% SDS-PAGE. For western blot analysis, an anti-CNF1 antibody (NG8, Santa Cruz Biotechnology, Dallas, TX, USA, diluted 1:1000) was used following the manufacturer’s instructions. 

CNF1 *wt* was obtained from the *E. coli* pISS392 strain (kindly provided by V. Falbo, Istituto Superiore di Sanità, Rome, Italy), and the plasmid coding for the recombinant protein CNF1 C866S, in which the enzymatic activity on Rho GTPases is abrogated by a change of cysteine with serine at position 866 [28], was kindly provided by E. Lemichez (U627 INSERM, Nice, France). Both toxins were purified essentially as described [29], with some modifications in the procedure. All dilutions were prepared with 20 mM Tris, pH 7.4.

### 4.2. Cell Cultures

HEp-2 laryngeal carcinoma cells and U87MG human GBM cells were grown in Dulbecco’s Modified Eagle’s Medium (D-MEM, EuroClone S.p.A., Pero, Italy) enriched with 10% fetal bovine serum (FBS, EuroClone), 100 U/l mL of penicillin and 100 μg/mL of streptomycin. The cells were grown in a controlled atmosphere incubator (5% CO2, 90% humidity, temperature 37 °C). For the various steps, cells were detached with 10 mM EDTA and 0.25% trypsin in PBS, without calcium and magnesium, at pH 7.4. All treatments were performed in a microbiological safety cabinet.

For all experiments, the cells were seeded in Petri dishes or in 24-well plates, depending on the type of assay: (i) 24-well plates for all titration, inhibition, and competition experiments; (ii) 60 mm Petri dishes for western blot. Cells were seeded at a density of 2 × 10^4^ cm^2^ cells. Twenty-four h after seeding, cells were treated with the An2-CNF1-H8, CNF1-H8 and *wt* CNF1 for different times depending on the experiment considered.

HBEC-5i cerebral microvascular endothelium cells (ATCC^®^ CRL3245™) were grown in 1% gelatin-coated culture flasks using DMEM:F12 with FBS 10% and 40 µg/mL endothelial growth supplement (Sigma–Aldrich, St. Louis, MO, USA). 

### 4.3. CNF1 Variant Titration 

Each variant was added by making successive dilutions in the culture medium, starting from a concentration of 10^−7^ M up to 10^−14^ M. After 24 h, HEp-2 cell multinucleation was estimated under a phase contrast microscope. At least 100 cells for each variant and for each dilution were counted, and the percentage of multinucleation was calculated. For HBEC-5i, since multinucleation is not evident, the cells were fixed in paraformaldehyde and labeled to highlight F-actin, which is significantly modified by CNF1, in terms of stress fibers and ruffles. All the toxins showed a similar activity ranging around 10^−10^ M. 

### 4.4. Competition Experiments with CNF1 C866S

HEp-2 cells were put on ice for 20 min. CNF1 C866S was added to the dilutions of 1:10, 1:100, 1:1000 for 10 min on ice. An2-CNF1-H8, CNF1-H8, and *wt* CNF1 were added at 10^−10^ M. After 30 min on ice, cells were washed 3 times with fresh medium before being placed in an incubator at 37 °C for 24 h. Finally, for both HEp-2 and HBEC-5i cells, the effect of the variants was measured.

### 4.5. Protein Extraction of Polyacrylamide and Western Blot Gel

One or 5 μL of the different variants were run on 8% or 12% polyacrylamide gel. After the run, the proteins were stained using the brilliant blue Comassie followed by discoloration. For western blot, proteins separated by polyacrylamide gel were transferred onto Polyvinylidene Fluoride (PVDF) membrane (Bio-Rad Laboratories, Inc., Hercules, CA, USA) and an anti-CNF1 antibody recognizing the C-terminal was used. For western blot, cells were washed twice with cold PBS and then lysed with a denaturing pad composed of 50 mmol/L Tris-HCl, pH 7.5, 2 mmol/L EDTA, 100 mmol/L NaCl, 1 mmol/L Na3Vo4, 1% Nonidet P-40, 10 g/mL leupeptin, 5 g/mL aprotinin, and 10 g/mL phenyl-methyl-sulfonyl fluoride for 10 min. The lysed cells were collected with a scraper, placed in the Eppendorf, and centrifuged for 5 min at 1200 rpm. The protein concentration of the supernatants was determined with the “Bio-Rad Protein Dc” assay (Bio-Rad Laboratories). Regards in vivo experiments, tissues were lysed in the same buffer, clarified and quantified as above described. Ten μg of protein per sample were taken and subsequently solubilized by boiling in “Laemmli Sample Buffer” (Sigma–Aldrich). The proteins were separated on SDS-PAGE polyacrylamide gel [30], and subsequently electrically transferred onto a PVDF membrane (Bio-Rad Laboratories) using a Semi-dry system (Bio-Rad Laboratories). According to this procedure, the membrane in contact with the gel is placed between two absorbent paper sheets damped with the Semi-dry buffer inside the transfer apparatus. After saturating the free membrane sites with Tris-buffered saline-tween 20 (TBS-T) (20 mM Tris-HCl pH 7.5, 150 mM NaCl, 0.02% Tween 20) containing 5% skimmed milk, the same membrane was incubated overnight at 4 °C with the following antigen-specific antibodies diluted in TBS-T containing 5% skimmed milk: anti-CNF1-NG8 (Abcam, Cambridge, UK, mouse monoclonal, diluted 1:1000); anti-GAPDH (Santa Cruz, mouse monoclonal antibody, diluted 1:2000); anti-Tom20 and anti-beta-gal (Cell Signaling Technology, Inc., Danvers, MA, USA, diluted 1:1000); anti-OPA-1 (BD bioscience, diluted 1:1000); anti-Bax (Cell Signaling, diluted 1:1000); anti-spinophilin (Upstate, Lake Placid, NY, USA, diluted 1:1000). 

Following washings in TBS-T, membranes were incubated with the species-specific HRP-conjugated secondary antibodies (Jackson ImmunoResearch, Ely, UK) and immune complexes were detected by chemiluminescent HRP substrates (Immobilion Western, Millipore, Burlington, MA, USA). The images were acquired through ChemiDoc (Bio-Rad Laboratories).

### 4.6. Cell Growth Experiments

U87MG cells were grown in 60 mm Petri dishes. Twenty-four h after seeding, cells were treated with the An2-CNF1-H8 and *wt* CNF1 at 3 different concentrations (10^−10^ M, 0.5 × 10^−9^, 0.25 × 10^−8^). At different time points, An2-CNF1-H8 and CNF1-treated cells were detached from the culture dish with 10 mM EDTA and 0.25% trypsin in PBS and counted by using a Neubauer chamber. Cell counts were conducted five times and at least three separate experiments were carried out.

### 4.7. Mouse Studies

C57BL6/J male mice (Charles River Italia), were housed under standard conditions (21/23 °C, 40/50% humidity, 12 h light-dark cycle, food and water ad libitum). All procedures were carried out in accordance with the European and National Legislation (63/2010 EU, DL 26/2014, respectively) and approved by the Italian Ministry of Health (n.469/2018-PR). Mice were treated by tail vein injection with An2-H8, CNF1-H8 (0.5 × 10^−10^ M in a final volume of 175 μL) or Tris. General health and behavior of each mouse were monitored for 10 days following *iv* injection. Then, mice were sacrificed, brain extracted from the skull, and brain regions rapidly dissected and frozen for further biochemical analyses.

### 4.8. Fluorescence Microscopy

U87MG control and treated cells were fixed and processed as previously described [21]. Fitc-phalloidin (Sigma–Aldrich) was used to stain F-actin and Mitotracker (Molecular Probes, Eugene, OR, USA) was used to stain mitochondria. Nuclei were stained with Hoechst 33258 (Sigma–Aldrich). Glass coverslips were observed with a fluorescence optical microscope Olympus BX51/BX52 and images were acquired using the program IAS 2000 (Delta System, Rome, Italy).

### 4.9. Statistical Analysis

Data are presented as mean ± sem. Statistical analysis was performed by one-way ANOVA. When a significant main effect was detected, Tukey’s test for post-hoc comparisons was performed, with *p* < 0.05 as a threshold for significant differences.

## Supplementary Materials

online at https://www.mdpi.com/2072-6651/12/5/291/s1, Figure S1: Hep-2 multinucleation, Figure S2: Fluorescence intensity, Figure S3: Spinophilin expression in hippocampal tissue.
