# Supplementary material for: Cnf1 Variants Endowed with the Ability to Cross the Blood–Brain Barrier: A New Potential Therapeutic Strategy for Glioblastoma
Source: Toxins (Basel). 2020 May 4;12(5):291. doi: 10.3390/toxins12050291 (PMC7290510; doi:10.3390/toxins12050291)
Supplement: Supplementary file 1 [file toxins-12-00291-s001.pdf]

Supplementary Materials: Cnf1 Variants Endowed with the Ability to Cross the Blood-Brain Barrier: A New Potential Therapeutic Strategy for Glioblastoma

Andrea Colarusso, Zaira Maroccia, Ermenegilda Parrilli, Elena Angela Pia Germinario, Andrea Fortuna, Stefano Loizzo, Laura Ricceri, Maria Luisa Tutino, Carla Fiorentini and Alessia Fabbri

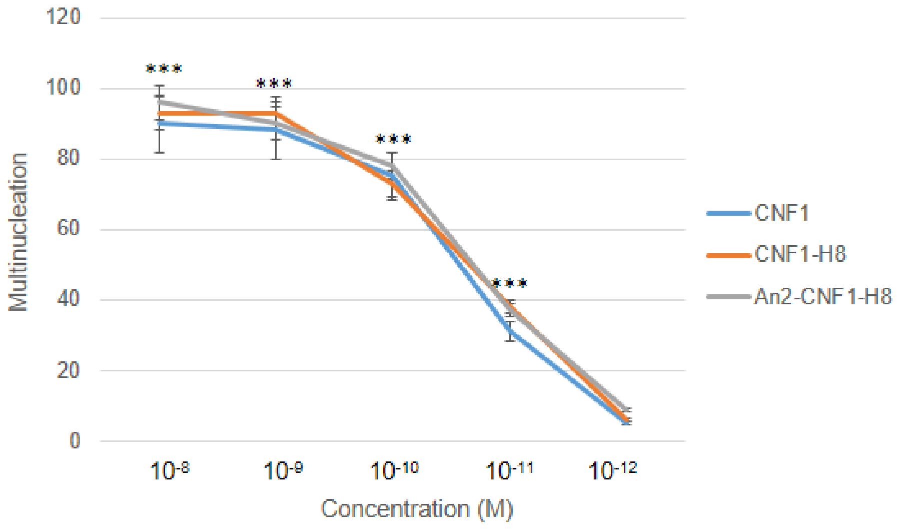

Figure S1. Hep-2 multinucleation. Graph showing the multinucleating effect on Hep-2 cells of decreasing doses of the different variants. *** $p < 0.001$.

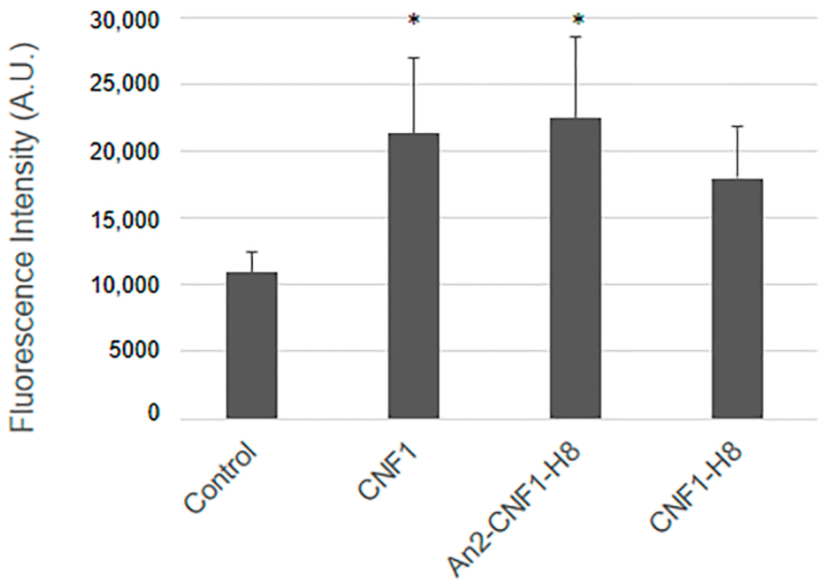

Figure S2. Fluorescence intensity. Graph showing the Fluorescence Intensity of F-actin staining on HBEC-5i cells. *** $p < 0.05$.

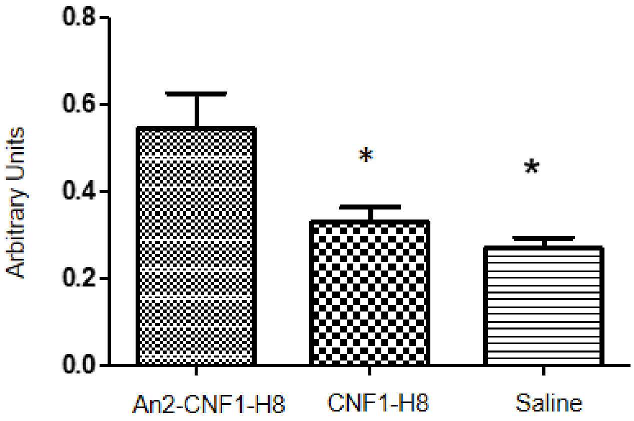

Figure S3. Spinophilin expression in hippocampal tissue. Graph showing Spinophilin protein expressions in hippocampal tissue (An2-CNF1-H8, *n* = 6; CNF1-H8, *n* = 4; Saline, *n* = 4)). The amounts of the above proteins are normalized as a function of α–tubulin. Note that An2-CNF1-H8 is able to increase spinophilin in hippocampus once intravenously inoculated. Values are expressed as mean ± SEM. * $p < 0.05$ for An2-CNF1-H8 vs other groups.
